# Spontaneous rescue from cystic fibrosis in a mouse model

**DOI:** 10.1186/1471-2156-7-18

**Published:** 2006-03-29

**Authors:** Nikoletta Charizopoulou, Martina Wilke, Martina Dorsch, Alice Bot, Huub Jorna, Silke Jansen, Frauke Stanke, Hans J Hedrich, Hugo R de Jonge, Burkhard Tümmler

**Affiliations:** 1 Klinische Forschergruppe, OE 6710, Medizinische Hochschule Hannover, D-30625 Hannover, Germany; 2 Department of Biochemistry, Erasmus University Medical Centre, PO Box 1738, 3000 DR Rotterdam, The Netherlands; 3 Zentrales Tierlaboratorium, OE 8600, Medizinische Hochschule Hannover, D-30625 Hannover, Germany

## Abstract

**Background:**

From the original *Cftr*^*TgH*(*neoim*)*Hgu *^mutant mouse model with a divergent genetic background (129P2, C57BL/6, MF1) we have generated two inbred *Cftr*^*TgH*(*neoim*)*Hgu *^mutant strains named CF/1-*Cftr*^*TgH*(*neoim*)*Hgu *^and CF/3-*Cftr*^*TgH*(*neoim*)*Hgu*^, which are fertile and show normal growth and lifespan. Initial genome wide scan analysis with microsatellite markers indicated that the two inbred strains differed on the genetic level. In order to further investigate whether these genetic differences have an impact on the disease phenotype of cystic fibrosis we characterised the phenotype of the two inbred strains.

**Results:**

Reduced amounts, compared to wild type control animals, of correctly spliced Cftr mRNA were detected in the nasal epithelia, lungs and the intestine of both inbred *Cftr*^*TgH*(*neoim*)*Hgu *^strains, with higher residual amount observed for CF/1-*Cftr*^*TgH*(*neoim*)*Hgu *^than CF/3-*Cftr*^*TgH*(*neoim*)*Hgu *^for every investigated tissue. Accordingly the amounts of wild type Cftr protein in the intestine were 9% for CF/1-*Cftr*^*TgH*(*neoim*)*Hgu *^and 4% for CF/3-*Cftr*^*TgH*(*neoim*)*Hgu*^. Unlike the apparent strain and/or tissue specific regulation of Cftr mRNA splicing, short circuit current measurements in the respiratory and intestinal epithelium revealed that both strains have ameliorated the basic defect of cystic fibrosis with a presentation of a normal electrophysiology in both tissues.

**Conclusion:**

Unlike the outbred *Cftr*^*TgH*(*neoim*)*Hgu *^insertional mouse model, which displayed the electrophysiological defect in the gastrointestinal and respiratory tracts characteristic of cystic fibrosis, both inbred *Cftr*^*TgH*(*neoim*)*Hgu *^strains have ameliorated the electrophysiological defect. On the basis of these findings both CF/1-*Cftr*^*TgH*(*neoim*)*Hgu *^and CF/3-*Cftr*^*TgH*(*neoim*)*Hgu *^offer an excellent model whereby determination of the minimal levels of protein required for the restoration of the basic defect of cystic fibrosis can be studied, along with the modulating factors which may affect this outcome.

## Background

Cystic fibrosis (CF) is a severe autosomal recessive disorder characterised by defective chloride transport in epithelial cells and excess mucus secretion. It is caused by mutations in the cystic fibrosis transmembrane conductance regulator gene (*CFTR*), resulting in defective cAMP-dependent chloride conductance [1].

Isolation of the mouse *Cftr *gene enabled the modelling of the disease in the mouse by gene targeting in embryonic stem cells. Dorin et al [2] described a transgenic mouse model *Cftr*^*TgH*(*neoim*)*Hgu*^/*Cftr*^*TgH*(*neoim*)*Hgu*^, generated following targeted insertional mutagenesis into exon 10 of the murine *Cftr *gene in embryonic stem cells. Unlike the *Cftr *mutants created by gene replacement low levels of wild type Cftr mRNA were produced as a result of exon skipping and aberrant splicing [3]. In the human, *CFTR *mutations affecting the pre-mRNA splicing of the gene can also generate both aberrant and correct transcripts, the level of which varies among patients and among organs of the same patient [4-8]. Patients carrying such mutations show variability of disease expression, which has been found to be inversely correlated with the levels of correctly spliced transcripts, such that lower levels are associated with severe disease and higher levels with milder phenotype, suggesting a role for splicing regulation as a genetic modifier [9]. The original *Cftr*^*TgH*(*neoim*)*Hgu*^/*Cftr*^*TgH*(*neoim*)*Hgu *^mouse suffered from only mild intestinal obstruction, but it exhibited typical pathophysiological features of CF in gut, lung and reproductive tract [2,10]. *Cftr*^*TgH*(*neoim*)*Hgu*^/*Cftr*^*TgH*(*neoim*)*Hgu *^mutant mice displayed the electrophysiological defect in the gastrointestinal and respiratory tract characteristic of CF and could be unequivocally distinguished from their non-cf littermates (*Cftr*^*TgH*(*neoim*)*Hgu*^/+ and +/+) on this basis [2,11]. The residual amounts of correctly spliced mRNA apparently led to the production of some functional Cftr protein that was sufficient to ameliorate the otherwise fatal intestinal obstruction but not enough to ameliorate the intestinal electrophysiological disease phenotype [3].

From the original *Cftr*^*TgH*(*neoim*)*Hgu *^mutant mouse model [2] with a divergent genetic background we have generated two inbred *Cftr*^*TgH*(*neoim*)*Hgu *^mutant lines named CF/1-*Cftr*^*TgH*(*neoim*)*Hgu *^and CF/3-*Cftr*^*TgH*(*neoim*)*Hgu *^using brother × sister mating for more than 30 generations. Phenotypic evaluation of the inbred CF/1-*Cftr*^*TgH*(*neoim*)*Hgu *^and CF/3-*Cftr*^*TgH*(*neoim*)*Hgu *^animals indicated that both strains have ameliorated the basic defect of CF with a presentation of a normal electrophysiology in both the intestinal and nasal epithelium. Unlike leaky splicing mutations in the human the reduced amounts of correctly spliced mRNA and the subsequent low amounts of wild type Cftr protein substantiated for the restoration of the disease phenotype in the two inbred *Cftr*^*TgH*(*neoim*)*Hgu *^strains. Based on this finding both CF/1-*Cftr*^*TgH*(*neoim*)*Hgu *^and CF/3-*Cftr*^*TgH*(*neoim*)*Hgu *^offer an excellent model to determine the minimal levels of wild type Cftr required to ensure a normal chloride secretory capacity of the intestine preventing intestinal obstruction.

There do exist a number of case reports in the literature of how a deleterious mutation is partially corrected by the affected patient. First, the interaction of drugs and/or proteins with sequences in the 3' untranslated region may lead to stabilisation of the mRNA transcript [12]. Second, nonsense-associated altered splicing is a putative correctional response that up-regulates alternatively spliced transcripts that have skipped offending premature termination codons. Third, aminoterminal located nonsense mutations may be partially rescued by the expression of the partially functional amino-truncated protein generated from an alternative in-frame translation start site. These attenuation mechanisms have been reported for mild forms of non-Herlitz junctional epidermolysis bullosa [13], the laryngo-onycho-cutaneous syndrome [14] and X-linked adrenal hypoplasia congenita [15]. Our CF mouse model is a prototype of how a genuine lethal genetic predisposition can be spontaneously bypassed or compensated by post-transcriptional rescue.

## Results

### Generation of the inbred CF/1-*Cftr*^*TgH*(*neoim*)*Hgu *^and CF/3-*Cftr*^*TgH*(*neoim*)*Hgu *^mutant mice

Dorin et al described in 1992 [2] the generation of the transgenic CF mouse model *Cftr*^*TgH*(*neoim*)*Hgu*^/*Cftr*^*TgH*(*neoim*)*Hgu*^. Targeted insertional mutagenesis into exon 10 of the murine *Cftr *gene was performed in E14 embryonic stem cells (129P2 background). Correctly targeted clones were injected into C57Bl/6 blastocysts. Male chimeric mice were mated to outbred HsdOla:MF1 females [2].

In case of the Hannover pedigree, the male chimaera 40 was crossed with one MF1 female. The sibpair 40.15 from this mating both mice of which were heterozygous 129P2/MF1 (*Cftr*^*TgH*(*neoim*)*Hgu*^/+) became the founder breeding pair (generation F_0_) for the Hannover mouse lines. Transgenic *Cftr*^*TgH*(*neoim*)*Hgu*^/*Cftr*^*TgH*(*neoim*)*Hgu *^littermates were subjected to brother – sister mating. By generation F_4 _(that was sent to Hannover) the same *Cftr*^*TgH*(*neoim*)*Hgu*^/*Cftr*^*TgH*(*neoim*)*Hgu *^male was mated with two *Cftr*^*TgH*(*neoim*)*Hgu*^/*Cftr*^*TgH*(*neoim*)*Hgu *^female littermates. Two littermates each from each litter became the founders of the two individual inbred *Cftr*^*TgH*(*neoim*)*Hgu *^strains CF/1-*Cftr*^*TgH*(*neoim*)*Hgu *^and CF/3-*Cftr*^*TgH*(*neoim*)*Hgu *^which were generated by strict brother-sister mating of *Cftr*^*TgH*(*neoim*)*Hgu*^/*Cftr*^*TgH*(*neoim*)*Hgu *^homozygous mice for now more than 30 generations.

### Genome wide microsatellite genotyping

CF/1-*Cftr*^*TgH*(*neoim*)*Hgu *^and CF/3-*Cftr*^*TgH*(*neoim*)*Hgu *^mice of generation F_26 _were typed at a 20 cM spacing with microsatellite markers to assess their genome-wide status of homozygosity and mutual genetic diversity. The criterion for the selection of the first 78 markers was their informativity to differentiate between the three reference strains BALB/c, C57BL/6 and DBA/2. Informative loci between the two inbred CF strains were genotyped with flanking markers in order to map the regions of genetic heterogeneity, bringing this to a total of 105 microsatellite markers.

Sixteen out of the 105 microsatellites were polymorphic between CF/1-*Cftr*^*TgH*(*neoim*)*Hgu *^and CF/3-*Cftr*^*TgH*(*neoim*)*Hgu *^[see Additional File 1] indicating that these two strains are not identical to one another at all loci. Residual heterozygosity was detected for eight microsatellites [see Additional File 1] three of which were segregating in both CF strains. CF/1-*Cftr*^*TgH*(*neoim*)*Hgu *^and CF/3-*Cftr*^*TgH*(*neoim*)*Hgu *^differ from C57BL/6, BALB/c and DBA/2 strains in 58, 52 and 58 of the 105 markers, respectively [see Additional File 1]. In summary, the two CF inbred strains are highly similar to one another differing in eight chromosomal regions, but they deviate significantly from the three common inbred laboratory strains C57BL/6, DBA/2 and BALB/c.

### Cftr mRNA analysis

In order to determine whether CF/1-*Cftr*^*TgH*(*neoim*)*Hgu *^and CF/3-*Cftr*^*TgH*(*neoim*)*Hgu *^mutant animals produce correctly spliced wild type Cftr mRNA as previously described for their progenitor with a mixed genetic background [2,3] we used RT-PCR spanning exon 9 to exon 13 encompassing the vector insertion site, using wild type MF1 mouse as control. Correctly spliced wild type Cftr mRNA corresponded to a cDNA product of 555 bp in size. Sequencing of the 555 bp PCR products obtained for the CF/1-*Cftr*^*TgH*(*neoim*)*Hgu *^mutant mice (ileum) revealed that the observed reduced amounts correspond 100% to wild type Cftr cDNA sequence. Moreover, a fragment of approximately 750 bp was detected in samples of homozygous CF mutants but not in wild type animals. Sequencing of this product demonstrated that it corresponded to the previously described [3] alternatively spliced mRNA encompassing the 206 bp fragment from the targeting vector pIV3.5H spliced between exon 9 and exon 10. However, this alternatively spliced mutant mRNA unlike in the original report [3] was not consistently detected.

The amounts of total and correctly spliced wild type Cftr mRNA in lungs and intestine were determined at different stages of the inbreeding procedure (Table 1). The total amount of Cftr mRNA did not significantly change over time in lungs and intestine and was about 1- to 4-fold lower than in the corresponding tissues from Ztm:MF1 outbred mice (Table 1). The absolute and relative amounts of wild type Cftr mRNA, however, decreased during inbreeding. CF/1-*Cftr*^*TgH*(*neoim*)*Hgu *^mice of generations F_8 _– F_10 _had 7–8% of their Cftr mRNA correctly spliced, whereas ten generations later the F_18 _mice were carrying about 5% or 3% wild type Cftr mRNA in their intestine and lungs, respectively (Table 1). In F_18 _CF/3-*Cftr*^*TgH*(*neoim*)*Hgu *^mice the amount of correctly spliced Cftr mRNA had decreased below 1% in the intestine and was at the limit of detection by RT/PCR (<< 0.1%) in the lungs (Table 1). After we had detected Cftr immunoreative protein signals of differential intensity in the intestinal and upper respiratory epithelia (see below), we reinvestigated the contents of correctly spliced Cftr mRNA in F_32 _CF mice. The levels of wild type Cftr mRNA in nasal epithelium were determined to be 15% (CF/1-*Cftr*^*TgH*(*neoim*)*Hgu*^) and 2% (CF/3-*Cftr*^*TgH*(*neoim*)*Hgu*^) of those of outbred Ztm:MF1 mice. MF1 expressed more Cftr in the intestine than in the nose; the relative levels decreased from duodenum to jejunum and ileum. The pposite gradient was observed in the F_32 _CF mice. No correctly spliced Cftr mRNA was detectable in the duodenum of both CF/1-*Cftr*^*TgH*(*neoim*)*Hgu *^and CF/3-*Cftr*^*TgH*(*neoim*)*Hgu*^. The levels of wild type Cftr mRNA in jejunum and ileum of CF/1-*Cftr*^*TgH*(*neoim*)*Hgu *^mice, however, were about 4% and close to 10% of total Cftr mRNA, respectively. Consistent with data from the F_18 _mice, CF/3-*Cftr*^*TgH*(*neoim*)*Hgu *^showed about five- to tenfold lower levels of correctly spliced Cftr mRNA in jejunum and ileum, respectively than CF/1-*Cftr*^*TgH*(*neoim*)*Hgu *^mice.

**Table 1:** Cftr mRNA expression in CF/1-*Cftr*^*TgH*(*neoim*)*Hgu *^and CF/3-*Cftr*^*TgH*(*neoim*)*Hgu *^mice

	Log (molecules Cftr mRNA)/μg RNA		wild-type/total Cftr mRNA [%]
	Total Cftr mRNA^a^	wild-type Cftr mRNA^b^	
F_8 _– F_10 _CF/1-*Cftr*^*TgH*(*neoim*)*Hgu *^(n = 10)			
Lung	4.7 ± 0.3	3.6 ± 0.3	7.9 ± 2.1
Intestine	5.1 ± 0.2	4.0 ± 0.2	7.1 ± 1.6
			
F_18 _CF/1-*Cftr*^*TgH*(*neoim*)*Hgu *^mice (n = 6)			
Lung	4.5 ± 0.3	2.9 ± 0.6	2.7 ± 1.9
Intestine	5.3 ± 0.2	4.0 ± 0.2	5.3 ± 1.3
			
F_10 _CF/3-*Cftr*^*TgH*(*neoim*)*Hgu *^mouse (n = 1)			
Lung	4.5	3.1	4
Intestine	5.0	3.6	4
			
F_18 _CF/3-*Cftr*^*TgH*(*neoim*)*Hgu *^mice (n = 6)			
Lung	4.9 ± 0.2	< 2	<< 0.1
Intestine	5.1 ± 0.2	3.0 ± 0.5	0.8 ± 0.5
			
Ztm:MF1 mice (positive control, n = 4)			
Lung	5.1 ± 0.2	5.1 ± 0.2	
Intestine	5.3 ± 0.1	5.3 ± 0.1	

Reference: log (molecules aldolase mRNA)/μg RNA = 7.26 ± 0.12

^a^cDNA amplified by PCR with mmcftr primer pair A1 – A2;

^b^cDNA amplified by PCR with mmcftr primer pair B1 – B2 (see Methods section)

### Cftr processing in CF/1-*Cftr*^*TgH*(*neoim*)*Hgu *^and CF/3-*Cftr*^*TgH*(*neoim*)*Hgu *^mutant mice

**Figure 1:**
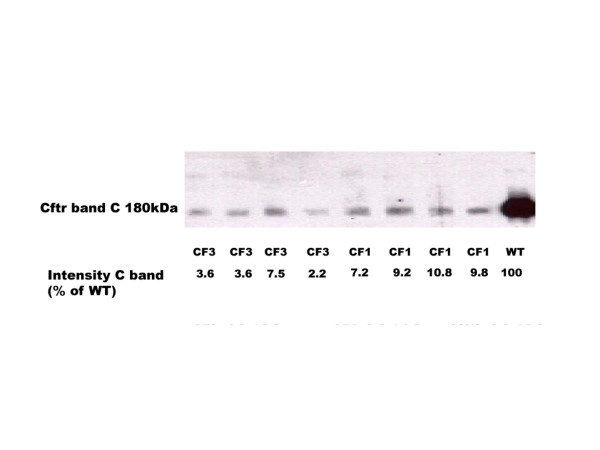
Quantification of wild type Cftr protein in apical membranes (only band C present) of enterocytes of F_28 _CF/1-*Cftr*^*TgH*(*neoim*)*Hgu *^(n = 4) and F_27 _CF/3-*Cftr*^*TgH*(*neoim*)*Hgu *^(n = 4) mutant mice. Blots were labelled using the Cftr specific R3195 antibody.

Analysis of brush border membrane vesicle (BBMV) preparations consisting of apical membranes of mutant homozygous F_27 _– F_30 _CF/1-*Cftr*^*TgH*(*neoim*)*Hgu *^(n = 4) and CF/3-*Cftr*^*TgH*(*neoim*)*Hgu *^mice (n = 4) demonstrated the presence of fully glycosylated isoform (band C) of Cftr in the small intestine of these mice (Figure 1). The intensity of the C band of these mice was reduced when compared with the intensity of the C band of the wild type MF1 mice (n = 2). By running individual samples of the CF/1-*Cftr*^*TgH*(*neoim*)*Hgu *^and CF/3-*Cftr*^*TgH*(*neoim*)*Hgu *^mutant mice together with serial dilutions (5×, 10×, 20×, 50×, 100×) of membranes from the MF1 wild type mice the amount of mature CFTR (band C) in the two inbred mutant mice was estimated to be 9.2 ± 1.5% of wild type for CF/1-*Cftr*^*TgH*(*neoim*)*Hgu *^and 4.2 ± 2.3% of wild type Cftr protein for CF/3-*Cftr*^*TgH*(*neoim*)*Hgu*^.

### Phenotype of the inbred CF/1-*Cftr*^*TgH*(*neoim*)*Hgu *^and CF/3-*Cftr*^*TgH*(*neoim*)*Hgu *^mutant mice

Growth and life-span of homozygous CF/1-*Cftr*^*TgH*(*neoim*)*Hgu *^and CF/3-*Cftr*^*TgH*(*neoim*)*Hgu *^mice improved during inbreeding. Weight gain during the first months of life increased from 23 ± 1.3 g at 3 months for *Cftr*^*TgH*(*neoim*)*Hgu *^mice of generation 8 to more than 30 g at 3 months for mice of generation 30 (CF/1-*Cftr*^*TgH*(*neoim*)*Hgu*^: females 29 ± 4 g, males 36 ± 2 g; CF/3-*Cftr*^*TgH*(*neoim*)*Hgu*^: females 32 ± 3 g, males 31 ± 3 g; Ztm:MF1 controls: females 36 ± 3 g, males 43 ± 2 g). When we started inbreeding, perinatal mortality was observed that peaked at the time of weaning when 8–10% of litter died of intestinal obstruction. Survival was 76% at the age of 3 months for the F_8 _– F_10 _generations, and early deaths were still observed in the F_18 _generation. CF/1-*Cftr*^*TgH*(*neoim*)*Hgu *^and CF/3-*Cftr*^*TgH*(*neoim*)*Hgu *^mice of generation 25 or more, however, do not show any increased mortality over wild type animals (pre-or post weaning). Both males and females are fertile.

**Figure 2:**
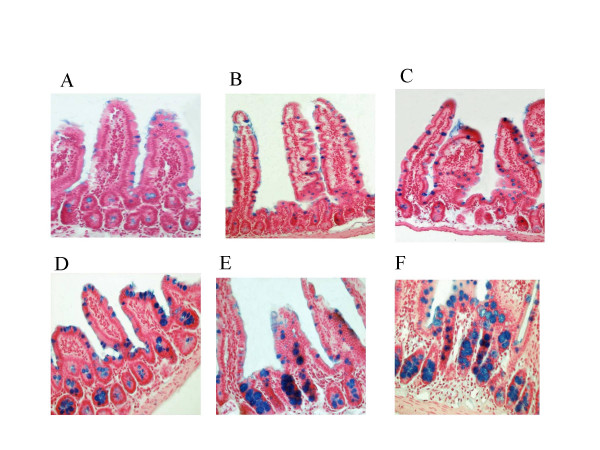
Alcian blue staining of deparaffinised sections of jejunum (A, B, C, magnification ×200) and ileum (D, E, F, magnification ×400) from wild type MF1 (A, D) and mutant F_28 _CF/1-*Cftr*^*TgH*(*neoim*)*Hgu *^(B, E) and F_27 _CF/3-*Cftr*^*TgH*(*neoim*)*Hgu *^(C, F) animals showed focal hypertrophy of goblet cells in the ileum of the two inbred *Cftr*^*TgH*(*neoim*)*Hgu *^mutant mice. Goblet cell counts are given as percentage of all epithelial cells. From each strain two mice and three sections/mouse were counted. Goblet cell count: CF/1 = 22.7% ± 2.3 (p = 0.112, p-value CF/1 compared to MF1), CF/3 = 23% ± 2.8 (p = 0.172, p-value CF/3 compared to MF1), MF1 = 16.8% ± 1.2. The jejunal sections of the same mice did not show any signs of goblet cell hypertrophy and were similar to wild type MF1 (Goblet cell count: CF/1 = 7.4% ± 0.4 (p = 0.18, p-value CF/1 compared to MF1), CF/3 = 8.5% ± 1.2 (p = 0.77, p-value CF/3 compared to MF1), MF1 = 8.8% ± 0.7.

Histological analysis of intestinal sections from mice of generations 27 and 28 revealed focal hypertrophy of goblet cells in the CF/1-*Cftr*^*TgH*(*neoim*)*Hgu *^and CF/3-*Cftr*^*TgH*(*neoim*)*Hgu *^homozygous mutant mice in the ileum (Figure 2). However, this hypertrophy was not observed in sections obtained from the jejunum in both inbred *Cftr*^*TgH*(*neoim*)*Hgu *^strains (Figure 2).

### Cftr immunocytochemistry

**Figure 3:**
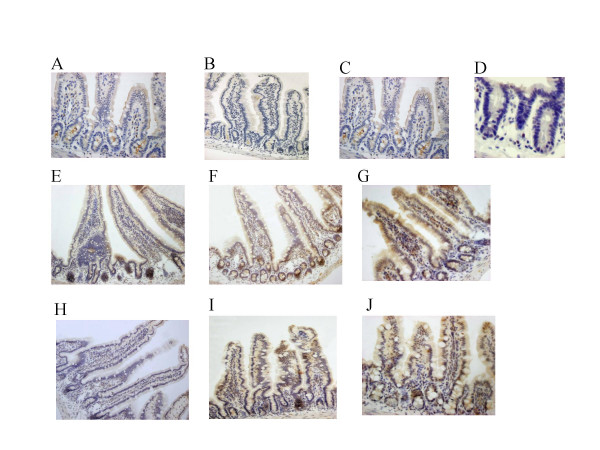
Immunohistological analysis of *Cftr*^*TgH*(*neoim*)*Hgu *^Cftr expression. Immunocytochemical staining of Cftr in the duodenum, jejunum, ileum of wild type mice MF1 (A, B, C), duodenum (E, magnification ×200), jejunum (F, magnification ×200) and ileum (G, magnification ×400) of homozygous F_28 _CF/1-*Cftr*^*TgH*(*neoim*)*Hgu *^and duodenum (H, magnification ×200), jejunum (I, magnification ×200) and ileum (J, magnification ×400) of F_27 _CF/3-*Cftr*^*TgH*(*neoim*)*Hgu *^mice was performed with the R3195 anti-Cftr antibody as described in Methods. Wild type mice showed intense Cftr staining of the apical border of epithelial cells. In CF/1-*Cftr*^*TgH*(*neoim*)*Hgu *^and CF/3-*Cftr*^*TgH*(*neoim*)*Hgu *^mutant mice Cftr staining was weaker and more diffuse mainly in the upper villus region. Ileum of a CF-Knock out mouse (D) was used as negative control.

In inbred F_28 _CF/1-*Cftr*^*TgH*(*neoim*)*Hgu *^staining with the R3195 Cftr antibody revealed weak and mainly diffuse signals with some apical labelling in the upper villus region of the ileum, whereas no specific immunoreactive signals could be detected in sections from duodenum and jejunum (Figure 3). Sections of the intestine (duodenum, jejunum, ileum) of F_27 _CF/3-*Cftr*^*TgH*(*neoim*)*Hgu *^mice showed substantially weaker staining at the limit of detection.

**Figure 4:**
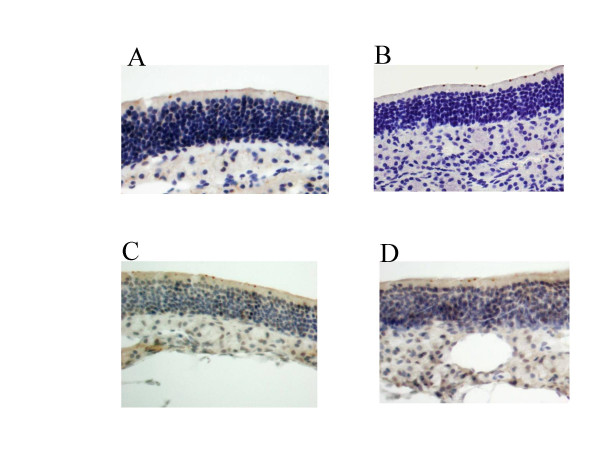
Cftr immunostaining in the nasal epithelium of wild type MF1 (A, magnification ×400), C57BL/6J (B, ×400), F_30 _CF/1-*Cftr*^*TgH*(*neoim*)*Hgu *^(C ×400), F_29 _CF/3-*Cftr*^*TgH*(*neoim*)*Hgu *^(D, ×400).

Cftr immunostaining of nasal respiratory epithelium (Figure 4) yielded strong punctuated apical labelling of similar intensity in F_30 _CF/1-*Cftr*^*TgH*(*neoim*)*Hgu*^, F_29 _CF/3-*Cftr*^*TgH*(*neoim*)*Hgu *^and wild type MF1 and C57BL/6J mice.

### Electrophysiological characteristics of CF/1-*Cftr*^*TgH*(*neoim*)*Hgu *^and CF/3-*Cftr*^*TgH*(*neoim*)*Hgu*^

**Figure 5:**
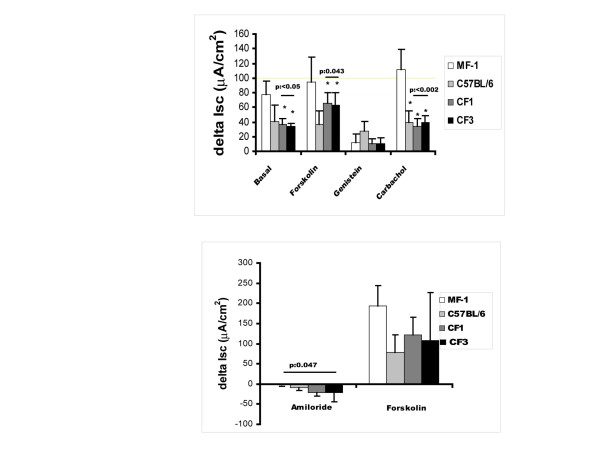
Short-circuit current (Isc) measurements. delta Isc represents the change in short-circuit current from baseline after addition of either forskolin, genistein, carbachol, or amiloride. Upper panel. Bioelectric characteristics of the F_28 _CF/1-*Cftr*^*TgH*(*neoim*)*Hgu *^(n = 4) and F_27 _CF/3-*Cftr*^*TgH*(*neoim*)*Hgu *^(n = 4) in the ileum compared with those of wild type MF1 (n = 5) and C57BL/6J (n = 4) mice. *, significant p-values compared to MF-1. Lower panel. Bioelectric characteristics of the F_28 _CF/1-*Cftr*^*TgH*(*neoim*)*Hgu *^and F_27 _CF/3-*Cftr*^*TgH*(*neoim*)*Hgu *^in the nasal epithelium compared with those of wild type MF1 and C57BL/6J. The amiloride response is significantly increased in both CF/1-*Cftr*^*TgH*(*neoim*)*Hgu *^and CF/3-*Cftr*^*TgH*(*neoim*)*Hgu *^mice compared to MF1 wild type outbred mice. C57BL/6J inbred wild type mice exhibited less chloride secretion than MF1. The forskolin evoked chloride secretion in both CF/1-*Cftr*^*TgH*(*neoim*)*Hgu *^*Cftr*^*TgH*(*neoim*)*Hgu *^and CF/3-*Cftr*^*TgH*(*neoim*)*Hgu *^was reduced compared to wild type MF1 but elevated compared to wild type C57BL/6J inbred mice.

The electrophysiological profile of F_28 _CF/1-*Cftr*^*TgH*(*neoim*)*Hgu *^and F_27 _CF/3-*Cftr*^*TgH*(*neoim*)*Hgu *^mice both in the nose and the intestine was examined by current measurements in Ussing chambers under short-circuit conditions. The chloride secretory responses of homozygous CF/1-*Cftr*^*TgH*(*neoim*)*Hgu *^and CF/3-*Cftr*^*TgH*(*neoim*)*Hgu *^CF mice were compared with those of inbred C57BL/6J and outbred MF1 non-CF mice. In the intestine, basal ion flow was similar in wild type C57BL/6J and all examined CF strains (Figure 5a) whereas MF1 exhibited a significantly higher response. The chloride secretory response of the two CF mutant strains evoked by forskolin amounted to about 60% of that observed in MF1 wild type animals and was higher than that of wild type C57BL/6J mice. The response to carbachol was indistinguishable between CF/1-*Cftr*^*TgH*(*neoim*)*Hgu *^and CF/3-*Cftr*^*TgH*(*neoim*)*Hgu *^and C57BL/6 wild type mice. Noticeably, MF1 mice (outbred stock) evoked a significantly higher response to carbachol when compared to wild type inbred C57BL/6J mice (almost 60% higher), indicative of an interstrain variation. In contrast genistein, which sustains the open state of CFTR channels [16,17], generated higher short-circuit current (Isc) currents in wild type C57BL/6J than in CF/1-*Cftr*^*TgH*(*neoim*)*Hgu *^and CF/3-*Cftr*^*TgH*(*neoim*)*Hgu *^mice.

In the nasal respiratory epithelium CF/1-*Cftr*^*TgH*(*neoim*)*Hgu *^and CF/3-*Cftr*^*TgH*(*neoim*)*Hgu *^mice exhibited a higher response after exposure to the sodium channel inhibitor amiloride in comparison with MF1 wild type, but not in comparison with C57BL/6J mice (Figure 5b). The forskolin activated cAMP-triggered chloride secretory response was indistinguishable from wild type C57BL/6J in CF/1-*Cftr*^*TgH*(*neoim*)*Hgu *^and CF/3-*Cftr*^*TgH*(*neoim*)*Hgu*^. The response of MF1 wild type mice was about two fold higher compared to inbred C57BL/6J wild type animals.

## Discussion

In contrast to mice with a complete disruption of the *Cftr *locus, inbred CF/1-*Cftr*^*TgH*(*neoim*)*Hgu *^and CF/3-*Cftr*^*TgH*(*neoim*)*Hgu *^mice like *Cftr*^*tm2Hgu *^and *Cftr*^*tm1Eur *^mutant mice do not suffer from intestinal blockage. Unlike their progenitor with a divergent genetic background [2,11], the two inbred CF strains managed to ameliorate the electrophysiological defect. The response of CF/1-*Cftr*^*TgH*(*neoim*)*Hgu *^and CF/3-*Cftr*^*TgH*(*neoim*)*Hgu *^mice to forskolin was indistinguishable from wild type C57BL/6J in the respiratory epithelia and intermediate between wild type C57BL/6J and MF1 control animals in the intestine. We envisage that during inbreeding modulating mechanisms were selected in these mice that influenced Cftr expression at the level of transcript, protein and function.

### Cftr splicing

Both novel inbred *Cftr*^*TgH*(*neoim*)*Hgu *^showed the ability to produce correctly spliced Cftr mRNA as previously reported for their outbred progenitor with a mixed genetic background in the respiratory and intestinal epithelia [3]. Interestingly, the total amount of Cftr mRNA did not change during inbreeding, but the levels of correctly spliced Cftr mRNA decreased to different extent in the two mutant inbred strains, with CF/1-*Cftr*^*TgH*(*neoim*)*Hgu *^showing higher levels for every investigated tissue. CF/1-*Cftr*^*TgH*(*neoim*)*Hgu *^and CF/3-*Cftr*^*TgH*(*neoim*)*Hgu *^were divergent in 16 out of 105 microsatellite genotypes in the genome-wide scan [18], verifying the genetic difference between the two novel *Cftr*^*TgH*(*neoim*)*Hgu *^inbred strains. This data suggests that interstrain genetic variation at non-*Cftr *loci could account for the phenotypic variation at the level of Cftr splicing observed between the two novel mutant strains.

In the intestine of both CF/1-*Cftr*^*TgH*(*neoim*)*Hgu *^and CF/3-*Cftr*^*TgH*(*neoim*)*Hgu *^the levels of Cftr expression are highest in the ileum and gradually decrease to lower levels in the jejunum and the duodenum. This tissue specific profile of Cftr expression, which is similar between CF/1-*Cftr*^*TgH*(*neoim*)*Hgu *^and CF/3-*Cftr*^*TgH*(*neoim*)*Hgu *^could be due to the existence of tissue specific elements which either down-regulate (duodenum, jejunum) or up-regulate (ileum, nasal epithelium) Cftr expression. Several splicing factors such as SRs and hnRNPs have been shown to modify the splicing pattern of human transcripts carrying splice mutations, overexpression of which can modulate the splicing pattern of *CFTR *alleles carrying splicing mutations [19-21], or affect tissue-specific control of CFTR expression [22]. Whether the tissue specific levels of Cftr expression depend on and/or are regulated by the levels of such splicing factors in these two strains remains to be investigated. Furthermore, in agreement with the previous study on the original *Cftr*^*TgH*(*neoim*)*Hgu *^mice with a mixed genetic background [3] the novel mRNA species that contained a 206 bp exon of vector sequence spliced between exon 9 and exon 10 was occasionally observed in both inbred strains. The 206 bp exon contains an in frame stop at a position less than half way into the message [3]. Such a premature stop codon may lead to exon skipping as has been demonstrated in several human genetic diseases [23-26] and could hence explain its inconsistent presence in samples from homozygous CF mutant mice.

### Cftr activity restoration: Efficient protein processing, trafficking and function

Regardless of the difference in correctly spliced Cftr mRNA levels both CF/1-*Cftr*^*TgH*(*neoim*)*Hgu *^and CF/3-*Cftr*^*TgH*(*neoim*)*Hgu *^mice synthesized and processed Cftr protein more efficiently in the ileum than wild type animals. Starting from small amounts of Cftr mRNA with wild type sequence, a higher proportion of protein was adequately folded, passed the ER quality control and was processed to complex-glycosylated ('band C') Cftr isoforms. Apparently the immature ER isoforms were more efficiently stabilised by molecular chaperones and protected from degradation than in normal mice.

The chloride secretory responses normalized to the pool of band C Cftr were 5- and 10-fold higher in CF/1-*Cftr*^*TgH*(*neoim*)*Hgu *^and CF/3-*Cftr*^*TgH*(*neoim*)*Hgu *^intestinal epithelia, respectively, than in wild type murine tissues. Several mechanisms could account for the higher ion flow per unit time and mass of mature Cftr in CF/1-*Cftr*^*TgH*(*neoim*)*Hgu *^and CF/3-*Cftr*^*TgH*(*neoim*)*Hgu *^mice. First, short-circuit current measurements are an indicator of transepithelial chloride transport and thus do not directly measure Cftr chloride channel activity. In wild type mice the transepithelial import of chloride is rate limiting, but at the 10–20 fold lower concentrations in our CF/1-*Cftr*^*TgH*(*neoim*)*Hgu *^and CF/3-*Cftr*^*TgH*(*neoim*)*Hgu *^mice the Cftr mediated current is the rate limiting step for chloride ion flow. At this lower density the relatively larger electrochemical driving force for the apical chloride exit will lead to relatively larger chloride flow/channel. In addition, efficient Cftr trafficking from the post-ER compartment to the apical membrane, along with a reduction in the lysosomal degradation may result in a higher proportion of mature protein localised in the apical membrane compared to the subapical compartments. Various components have been shown to influence maturation and trafficking of CFTR from the ER/Golgi compartment to the basolateral and apical compartments, i.e. COPI-coated vesicles [27], PDZ-containing proteins such as Csp [28], EBP50 [29,30], E3KARP [29], CAP70 [31], CAL [32], SNARE proteins [33], BAP31 [34] and numerous Rab proteins [35,36].

Third, the high ratio of forskolin to genistein Cl^- ^secretory responses suggests that even though there is a lower estimated channel density in the CF/1-*Cftr*^*TgH*(*neoim*)*Hgu *^and CF/3-*Cftr*^*TgH*(*neoim*)*Hgu *^compared to wild type in the apical membrane, the proportion of active channels, i.e. in an open state, is higher compared to wild type. The open probability of the CFTR channel is influenced by phosphorylation and ATP hydrolysis [37] and by the interaction with other proteins [38]. Phosphorylation of the regulatory domain is accomplished primarily by elevation of cAMP and activation of protein kinase A. Stronger adrenergic activation and higher levels of cAMP will sustain highly phosphorylated, more active CFTR channels. Moreover, since CFTR channel activity increases with the concentration of ATP [39], elevated intracellular ATP will allow for more CFTR-mediated chloride flux. In summary, higher yields of ion flow per unit mass mature Cftr can be achieved by the larger electrochemical driving force for the apical chloride exit and the modulation of a) posttranslational processing and trafficking, b) signalling and c) metabolic rate. The repeated cycles of inbreeding selected animals which were capable of producing sufficient litters and showed no severe CF disease symptoms. Hence, the rescue of CF to an almost normal phenotype in mice which remained genetically CF can be explained by the selection of genetic modifiers and adaptive mechanisms together with modulators operating at the transcriptional level. Our animal model is an extreme example of how a lethal genetic predisposition can be compensated.

### Comparison with the human

*CFTR *splice mutations that lead to the simultaneous presence of correctly and aberrantly spliced transcripts cause CF disease due to insufficient levels of the normal mRNA, and hence, functional protein. Among these are mutations leading to inclusion of intronic sequences, such as 3849+10 kb C-T [40], mutations leading to the skipping of *CFTR *exons, such as 2789+5 G-A [41], and the TG_m_-T_n _genotype at the *CFTR *intron 8 splice acceptor site [42-44].

Studies have reported a wide range of normal CFTR transcripts, from less than 4% to 20% [5,42,45] associated with "leaky" splicing mutations in the human. Disease severity also varies among individuals carrying these splicing mutations, ranging from healthy fertile individuals to the full presentation of classic CF. We know compound heterozygous 2789+5 G-A CF siblings of whom one is suffering from lung disease since adolescence, whereas the other enjoys normal daily life without any restrictions of physical activity still by the age of 60. An inverse correlation was found between the level of the correctly spliced mRNA and the severity of the disease [8,45]. In vitro studies in heterologous expression systems have shown that cellular factors such as hnRNP A1, ASF/SF2, SR proteins and TDP-43 that promote exon skipping and/or exon inclusion modulate the relative proportions of correctly to aberrantly spliced mRNA isoforms [21,46]. Moreover splicing is controlled by cis-regulatory enhancer and silencer elements part of which is under promoter control [6,47,48].

The major difference in the genotype – phenotype correlations of 'leaky' splice mutations between human individuals and the CF/1-*Cftr*^*TgH*(*neoim*)*Hgu *^and CF/3-*Cftr*^*TgH*(*neoim*)*Hgu *^mouse models resides in the fact that the manifestation of disease in humans is attenuated with increasing amounts of correctly spliced CFTR mRNA whereas the inbreeding of the mice selected for tissue specific modulation of lowered wild-type Cftr mRNA levels. Under the standardised conditions of the animal laboratory post-transcriptional mechanisms apparently gave rise to the rescue of the CF phenotype and the partial compensation of the basic defect. These yet undescribed factors that led to more efficient processing and higher channel activity of Cftr should also play a role for the modulation of disease in humans. The CF/1-*Cftr*^*TgH*(*neoim*)*Hgu *^and CF/3-*Cftr*^*TgH*(*neoim*)*Hgu *^mice are the adequate model to identify these beneficial modifiers that ameliorate the CF phenotype distal of the primary splicing defect.

## Conclusion

In this study we describe the phenotypic characterisation of two novel inbred cystic fibrosis mutant strains generated from an outbred colony of the original *Cftr*^*TgH*(*neoim*)*Hgu *^founder with a mixed genetic background (129P2, C57BL/6, MF1). Unlike their outbred progenitor low amounts of wild type Cftr protein substantiated for the amelioration of the disease phenotype in both CF/1-*Cftr*^*TgH*(*neoim*)*Hgu *^and CF/3-*Cftr*^*TgH*(*neoim*)*Hgu *^inbred mutant strains. The post-transcriptional rescue of disease-causing mutations is an emerging field of molecular medicine [49,50]. We envisage that both CF/1-*Cftr*^*TgH*(*neoim*)*Hgu *^and CF/3-*Cftr*^*TgH*(*neoim*)*Hgu *^can become excellent models for the identification of those mechanisms responsible of disease compensation and will increase our knowledge about genetic modifiers of inherited diseases in mammals.

## Methods

### Experimental animals

All experiments were approved by the local Institutional Animal Care and Research Advisory Committee as well as by the local government. *Cftr*^*TgH*(*neoim*)*Hgu *^were bred under specified pathogen-free conditions in the isolator unit of the Central Laboratory Animal Facility of the Hannover Medical School. Mice were kept in a flexible film isolator. The temperature within the insulator was maintained at 20–24°C with 40–50% relative humidity. Animals were fed an irradiated (50 kGy) standard chow (SSniff) and autoclaved water (134°C for 50 min) ad libitum. CFTR mRNA analyses were performed on Ztm:MF1, CF/1-*Cftr*^*TgH*(*neoim*)*Hgu *^(generations F_8_-F_10_, F_18_, F_32_) and CF/3-*Cftr*^*TgH*(*neoim*)*Hgu *^(generations F_10_, F_18_, F_32_) mice at the age of 3 and 6 months. Bioelectrics, Cftr immunoblot and immunocytochemistry were performed on female Ztm:MF1, CF/1-*Cftr*^*TgHeoim*)*Hgu *^(generations F_28_, F_30_) and CF/3-*Cftr*^*TgH*(*neoim*)*Hgu *^(generations F_27_, F_29_) mice at the age of 3 months.

CF/1-*Cftr*^*TgH*(*neoim*)*Hgu *^and CF/3-*Cftr*^*TgH*(*neoim*)*Hgu *^mice are available for the scientific community upon written request to HJH.

### Generation of inbred *Cftr*^*TgH*(*neoim*)*Hgu *^mutant mice

For the gene targeting and germline transmission to generate the *Cftr*^*TgH*(*neoim*)*Hgu *^mice, the embryonal stem cell line E14 was used [2]. E14 originates from the inbred mouse strain 129 (subline 129/Ola-Hsd, abbreviated designation: 129P2) [2]. These cells were injected into blastocysts of the inbred strain C57BL/6J. The resulting chimeric offspring was crossed to C57BL/6J and to the outbred strain MF1 for several times to increase reproduction [2]. By generation F_4 _the same *Cftr*^*TgH*(*neoim*)*Hgu*^/*Cftr*^*TgH*(*neoim*)*Hgu *^male was mated with two *Cftr*^*TgH*(*neoim*)*Hgu*^/*Cftr*^*TgH*(*neoim*)*Hgu *^female littermates. Two separate litters were obtained and two animals of each litter became the starting population for the establishment of the two individual inbred *Cftr*^*TgH*(*neoim*)*Hgu *^lines CF/1-*Cftr*^*TgH*(*neoim*)*Hgu *^and CF/3-*Cftr*^*TgH*(*neoim*)*Hgu *^which were generated by brother-sister mating for now more than 35 generations. Only one breeding pair per generation was utilized for the continuation of the line. In other words, littermates always shared the same ancestors. *Cftr *genotyping from tail or ear DNA extractions was conducted by Southern blot hybridization of XhoI/SalI-restricted genomic DNA with the *cftr *intron 10 probe 1.2 H [2].

### Genotyping of microsatellites

High molecular weight DNA was isolated from spleen tissue (from ten F_26 _CF/1-*Cftr*^*TgH*(*neoim*)*Hgu*^, ten F_26 _CF/3-*Cftr*^*TgH*(*neoim*)*Hgu*^, two BALB/c, two C57BL/6 and two DBA/2) [51]. The primers flanking the 105 chosen microsatellite regions were obtained from the MGI database [52]. Microsatellite markers were genotyped in 96 well plates purchased from Greiner, Frickenhausen, pre-coated with 50 ng DNA per well in a Hybaid Thermocycler (Hybaid, Teddington) with a heated lid. One of the two primers per microsatellite was 5'-terminal biotinylated (Invitrogen Ltd). PCR was performed for all microsatellites in a total volume of 30 μl, without oil overlay, using InViTaq polymerase (InViTek, Berlin) according to the instructions by the manufacturer. Following PCR an 8 μl aliquot was transferred to a multiwell plate and allowed to dry overnight at 37°C, dissolved in 10 μl loading buffer (0.2% w/v xylenecyanol and bromphenolblue in formamide) and denatured for 5 min at 95°C. The PCR products were separated by direct blotting electrophoresis (GATC 1500, MWG Biotech, Ebersberg, Germany) on a denaturing acrylamide gel (4% acrylamide/N,N'-methylenebisacrylamide 29:1 containing 6 M urea in 0.9 M Tris-0.9 M boric acid-0.02 M EDTA buffer) and simultaneously transferred to a Hybond N+membrane (Amersham). Signals were visualised by blocking the membrane in 1.5% (w/v) of blocking reagent in Buffer 1 (100 mM Tris-HCl, 150 mM NaCl, pH 7.5), followed by incubation in diluted solution of anti-biotin alkaline phosphatase conjugate in Buffer 1. The membrane was further washed three times with 1% Triton X-100 in Buffer 1 and equilibrated for 15 min in assay buffer (100 mM Tris-HCl, 100 mM NaCl, 50 mM MgCl_2_, pH 9.5). The membrane was covered for 5 min with reaction buffer containing 10%(v/v) Sapphire II (Tropix) and 600 μl CDPstar (Tropix) (12.5 mM) in 50 ml assay buffer, followed by rinsing with a solution containing 10% v/v Sapphire II and 60 μl CDPstar (12.5 mM) in 50 ml assay buffer. Signals were exposed to Kodak XA-R films. Evaluation of results was performed as described by Mekus et al [53].

### Isolation of RNA and quantitative RT/PCR

All tissue samples from Ztm: MF1 and F_8–10 _as well as F_18 _CF mice were immediately frozen in liquid nitrogen after extirpation and stored at -70°C until used. Prior to the isolation of RNA, all solutions and plasticware were treated for at least 12 h with 0.04% (by vol.) aqueous diethyl pyrocarbonate solution. RNA was isolated essentially as described by Chomczynski and Sacchi [54]. Briefly, tissue was ground with a pestle in a mortar under liquid nitrogen and then homogenized in denaturing solution (4 M guanidine thiocyanate, 25 mM sodium citrate, 0.5% sarcosyl, 200 mM β-mercaptoethanol). The homogenate was mixed sequentially with 1 M sodium acetate (pH 4), phenol and chloroform/isoamyl alcohol; the mixture was centrifuged and the RNA removed from the upper aqueous phase. Following isopropanol precipitation, the RNA pellet was redissolved in denaturing solution, reprecipitated with isopropanol, washed with ethanol, dried, dissolved in DEPC-treated water and stored at -70°C until used. The quality of the preparation was checked by UV spectrophotometry and ethidium bromide stain of 2 μg RNA separated by 1% agarose gel electrophoresis. The absence of contamination by DNA in the RNA preparation was verified by PCR with oald141N 5'-GGCAAGGGCATCCTGGCTGCAGA and oald581I 5'-TAACGGGCCAGAACATTGGCATT. These oligonucleotide primers yield a 421-bp product for both murine genomic DNA and reverse-transcribed murine RNA (PCR conditions per cycle: annealing at 62°C, synthesis at 72°C, denaturation at 92°C 60 s each).

For cDNA synthesis, 8 μg denatured RNA were added to the 40-μl reaction volume of 40 U avian myoblastoma virus reverse transcriptase, 120 μg/ml oligo(dT)_15_, 2000 U/ml RNasin, 0.9 mM dNTPs, 10 mM DTT, 75 mM KCl, 3 mM MgCl_2_, 50 mM Tris/HCl, pH 8.3, incubated for 1 h at 37°C, distributed in 10 μl aliquots and stored at -70°C for a maximum of one month. The differential amounts of total cftr mRNA and correctly spliced wild-type cftr mRNA transcripts in specimens from CF/1-*Cftr*^*TgH*(*neoim*)*Hgu *^and CF/3-*Cftr*^*TgH*(*neoim*)*Hgu *^mice were determined by quantitative PCR kinetics modifying a protocol described by Hoof et al [55]. Two oligonucleotide primer pairs A1–A2, B1–B2 were designed upstream of and encompassing the *neo *insertion site [2] (A1 mmcftr 1164–1189 5'-GCATTGTCCTACGTATGTCAGTCACG; A2 mmcftr 1524–1499 5'-CCTAGTCCAGTAGATCCAGTAATAGC; B1 mmcftr 1499–1524 5'-GCTATTACTGGATCTACTGGACTAGG; B2 mmcftr 2053–2032 5'-GCTCGGACGTAGACTTTGTAGC) (GenBank Accession Number: NM_021050).

Amplification of Cftr cDNA stretches by PCR with these primer pairs showed an identical kinetics of product formation (Δcmax/cycle: ± 0.4% intestine; ± 1.3% lungs) with Ztm:MF1 reverse-transcribed control RNA (PCR conditions per cycle for both primer pairs: annealing at 58°C, synthesis at 72°C, denaturation at 92°C 60 s each). For quantitation of mRNA, the kinetics of cDNA formation was monitored by incorporation of 1 × 10^5 ^Bq [α-^33^P]dATP (> 1 × 10^14 ^Bq/mmol) into cDNA. The PCR assay (50-μl reaction volume covered with 60 μl mineral oil) contained 0.5 μM of each oligonucleotide primer, 0.2 mM dNTP, 3 μl of oligoT-reverse transcribed reaction mix (see above), 1.3 μl DMSO, 1.5 U Taq DNA polymerase (InviTAQ), 7.5 mM MgCl_2_, 16 mM (NH_4_)_2_SO_4_, 50 mM Tris/HCl, pH 8.8, 0.1% Tween.

Aliquots of the same oligoT-reverse transcribed reaction mix were amplified in parallel in separate tubes in the same blocks of the thermocycler with primer pairs A1–A2, B1–B2 and oald141N-oald583I, respectively. During the exponential phase of the amplification of the respective cDNA aliquots of 10 μl were withdrawn in 2-cycle intervals and separated by 5% PAGE. The dried gel was exposed to a Fuji imaging plate type BAS-IIIs in order to quantify the yield of cDNA product by photoimaging signal. For each RNA preparation, the optimal window of PCR kinetics had been determined for each of the three cDNA sequences in a prior pilot PCR. For quantification of the cftr mRNA contents in the samples, data sets for each murine tissue were normalized to its invariant aldolase mRNA contents in order to adjust to the variable quality of mRNA template in the RNA preparations and assay-to-assay variability of the reverse transcription. This approach was verified by the observation that the repeated preparation of RNA from one tissue specimen showed similar variations in aldolase cDNA kinetics as tissue RNAs from unrelated animals. The quantitative amount of cftr mRNA in the various samples was calculated from the complete data set for one tissue whereby the experimental curves log [cDNA] vs. PCR reaction cycle n were converted into calibration curves cftr RNA molecules vs. PCR cycle for various yields of cDNA [55]. Only those datasets were evaluated that within experimental error had given indistinguishable Cftr cDNA formation kinetics with primer pairs A1–A2 and B1–B2 for reverse-transcribed Ztm:MF1 RNA (positive control). Cftr mRNA contents of each RNA preparation was assayed at least in triplicate.

In case of the analysis of F_32 _CF/1-*Cftr*^*TgH*(*neoim*)*Hgu *^and F_32 _CF/3-*Cftr*^*TgH*(*neoim*)*Hgu *^animals the tissues (duodenum, jejunum, ileum, nose) were stored in RNAlater (Ambion) after extirpation and stored at -20°C until use. RNA was extracted using the RNAeasy mini kit (Qiagen) following the instructions of the manufacturer. Extracted RNA was stored at -80°C until use. The amount of aldolase and correctly spliced Cftr mRNA was determined by semiquantitative RT/PCR kinetics. We titrated for the first reaction cycle, when the gel-separated cDNA became visible by ethidium bromide fluorescence during the late exponential phase of PCR [56]. This procedure yielded an estimate of mRNA concentration within half an order of magnitude, whereas the interexperimental error of the quantitative PCR on mRNA contents in F_8–10 _and F_18 _CF mice that measured the incorporation of labelled nucleotide during the exponential phase of PCR was twofold or less.

### Sequencing

Following RT-PCR cDNA products were sequenced by Qiagen GmbH.

### Western blot analysis

Wild type MF1 mice and CF/1-*Cftr*^*TgH*(*neoim*)*Hgu *^and CF/3-*Cftr*^*TgH*(*neoim*)*Hgu *^mutant mice were anaesthetized with a hypnorm/diazepam mixture. Their abdomens were opened and their small intestines dissected. Epithelial cells originating principally from the villus region were isolated at 0–4°C from the jejunum by everting the intestinal segments on metal rods attached to a vibration apparatus (Vibromixer type E1 from Chemap A.G.) and exposing them to vibration (50 Hz, amplitude 1.5 mm) for 30 min in 0.14 M NaCl containing 5 mM EDTA pH 7.4. Detached jejunal enterocytes were collected by centrifugation at 800 g for 15 s and suspended in 10 ml of a medium containing 12 mM Tris-HCl pH 7.4, 0.3 M mannitol, 10 mM KCl, 0.5 mM EDTA and a protease-inhibitor cocktail containing 0.3 mM Pefablock (La Roche), 10 μg/ml aprotinine, 5 μg/ml leupeptine, 1 μg/ml pepstatin A, 1 μg/ml chymostatin, 50 μg/ml soybean trypsin inhibitor and 0.03 g/l phosphoramidon. Vesiculation of intestinal membranes was achieved by a freeze-thaw procedure described initially for rat enterocytes and crude microsomal membranes were isolated from half of the cell lysate by a two-step differential centrifugation procedure (10 min, 4000 g, followed by 60 min, 40 000 g). The other half was used to isolate BBMV by differential precipitation with 10 mM MgCl_2 _and differential centrifugation (15 min, 3000 g followed by 30 min, 27 000 g) essentially as described by [57]. The membrane pellets were solubilized by vortexing in 30 μl modified Laemmli sample buffer [0.06 M Tris-HCl; 2% (w/v) SDS, 10% (w/v) glycerol, 0.1 M dithiothreitol, 0.1% (w/v) bromophenol blue and the protease inhibitor cocktail, pH 6.8] and incubated for 30 min at room temperature. Following centrifugation (2 min, 8000 g) samples of the supernatant (10 μl, adjusted to 20 μg protein) were separated on 6% polyacrylamide slab gels using a Bio-Rad Miniprotean apparatus (Bio-Rad Laboratories). Proteins were subsequently electroblotted onto nitrocellulose paper (0.1 μm pore size; Schleicher and Schuell) in 0.025 M Tris, 0.192 M glycine, 20% (v/v) methanol. The blots were incubated overnight at 4°C with 0.02 M Tris-HCl, 0.15 M NaCl, 0.1% (w/v) Tween 20 pH 7.5 (TTBS), followed by overnight incubation at 4°C with a 1:1000 dilution of affinity-purified anti-CFTR antibody R3195 in TTBS. Blots were washed three times in TTBS, incubated with peroxidase-conjugated anti-rabbit IgG (Tago Inc.; 1:3000 in TTBS for 2 h), and washed four times with TTBS. Peroxidase activity was detected with bioluminescence reagent (ECL kit; Amersham) on X-ray film, and CFTR bands were quantitated with the Molecular Imaging System GS-363 (Bio-Rad).

### CFTR antibody

The rabbit polyclonal antibody R3195 was raised against a thyroglobulin-conjugated 13 amino acid COOH-terminal peptide sequence of rodent CFTR and was affinity purified on a peptide epoxide-activated Sepharose column, eluted with 4.9 M MgCl_2_, dialysed and concentrated. CFTR labelling specificity has been demonstrated previously in western blot and immunocytochemical assays by the loss of immunostaining in tissue specimens from CFTR^-/- ^mice [58].

### Immunocytochemical analysis

Intestine: Wild type (MF1 and C57BL/6J) and mutant CF/1-*Cftr*^*TgH*(*neoim*)*Hgu *^and CF/3-*Cftr*^*TgH*(*neoim*)*Hgu *^mice were sacrificed by cervical dislocation, the intestine was dissected and the jejunum, duodenum and ileum were rinsed with ice-cold saline and fixed in 3% (w/v) paraformaldehyde for 16 h, prior to standard paraffin embedding. Sections (5 μm) were deparaffinised, followed by microwave treatment in 0.01 M sodium citrate solution according to Devys et al. [59]. Endogenous peroxidase activity was blocked by a 30 min pre-incubation in 0.1 M PBS, 0.6% (v/v) H_2_O_2 _and 0.12% (w/v) sodium azide. Subsequently, sections were incubated with antibody R3195 (1:100) at room temperature for 1.5 h followed by a 60 min incubation with a peroxidase-conjugated secondary antibody. Enzymatic detection of antigen-antibody complexes was achieved by incubation in substrate solution containing H_2_O_2 _and 3,3'-diaminobenzidine tetrahydrochloride (DAB; Serva). Finally, the sections were counterstained with haematoxylin. Labelling specificity was verified by incubations without primary antibody. In both cases, background labelling appeared negligible.

Nasal: Freshly excised nasal tissue from wild type MF1 and C57BL/6J and mutant CF/1-*Cftr*^*TgH*(*neoim*)*Hgu*^, CF/3-*Cftr*^*TgH*(*neoim*)*Hgu *^mice was used for immunocytochemical analysis. The tissue was treated as described for the intestine.

### Histological analysis

Deparaffinised sections (5 μm) were stained with Alcian blue (1% w/v in 3% v/v acetic acid, Sigma) for 30 min at RT, rinsed in tab water and counterstained with Nuclear fast red (Sigma) for 10 min.

### Short-circuit current measurements

Freshly excised mouse ileum and nasal epithelium of CF/1-*Cftr*^*TgH*(*neoim*)*Hgu *^and CF/3-*Cftr*^*TgH*(*neoim*)*Hgu *^homozygous animals were used for short-circuit current (Isc) measurements and compared with wild type controls. All experiments were done in duplicate, n = the number of mice used. Experiments were performed at 37°C. The basic perfusion solution (modified Meyler's solution) consisted of 105 mM NaCl, 4.7 mM KCl, 1.3 mM CaCl_2_, 1.0 mM MgCl_2_, 20.2 mM NaHCO_3_, 0.4 mM NaH_2_PO_4_, 10 mM HEPES, saturated with 95% O_2 _and 5% CO_2_, pH 7.4.

Mouse ileum was excised under hypnorm/diazepam anaesthesia and reverted on a plastic rod. The muscle layer was cut longitudinally using a blunt razor blade and was stripped of fat manually. The stripped tissue was mounted in a holder with the mucosal side up (exposed tissue area 0.2 cm^2^). After insertion of the holder into the Ussing chamber the tissue was allowed to recover for 10–20 min and to reach a stable baseline. To the serosal side of the ileum glucose (10 mM) and indomethacin (10 mM) were added. After equilibrium, the following compounds were added consecutively to the mucosal (M) or serosal (S) side of the tissue: Forskolin (10 μM, S), genistein (100 μM, M+S) and carbachol (200 μM, S). All compounds were present throughout the experiment.

Mouse nasal epithelium was isolated as described by Grubb et. al. [60]. In brief, the mice were sacrificed by cervical dislocation and the skin of the head was peeled back in order to reach the underlying paired nasal bones. These were removed and the two sheets of the nasal epithelia, separated by the septum, were isolated independently. The sheets of epithelia were mounted immediately between the Ussing chambers (exposed area 1.13 mm^2^). The chambers were filled with gassed modified Meyler's solution supplemented with glucose (10 mM) and indomethacin (10 mM) and the short circuit measurements were started. After equilibration amiloride (10 μM, M) was added followed by forskolin (10 μM, after stabilization of the current).

### Statistics

P-values were determined by using the student's t-test. P-values < 0.05 were considered significant.

## Authors' contributions

NC performed the genome-wide scan with microsatellite markers, SJ and NC executed RNA isolations and performed RT-PCR experiments, MW carried out all short-circuit current measurements, MD participated in the supervision of the animal breeding, AB performed western blot analysis, HJ carried out the immunocytochemical and histological analysis, HJH designed and supervised all animal breeding, FS set up all PCR-related methodology and quality control and BT revisited the primary genetic and phenotypic murine data gathered at the Hannover site since 1994. HRDJ and BT conceived and supervised the study and participated in the design of experiments and result analysis. All authors contributed to the writing of this manuscript.

## Additional Files

File name: Microsatellites Additional File 1.pdf

File Type: PDF

File Size: 279 kb

Title of dataset: Marker genotypes of 105 microsatellites of F_26 _CF/1-*Cftr*^*TgH*(*neoim*)*Hgu *^and F_26 _CF/3-*Cftr*^*TgH*(*neoim*)*Hgu *^CF mice and the reference inbred strains C57BL/6, BALB/c, DBA/2J.

Description. CF/1-*Cftr*^*TgH*(*neoim*)*Hgu *^and CF/3-*Cftr*^*TgH*(*neoim*)*Hgu *^mice of generation F_26 _were typed at a 20 cM spacing with microsatellite markers. The criterion for the selection of the first 78 markers was their informativity to differentiate between the three reference strains BALB/c, C57BL/6 and DBA/2. Informative loci between the two inbred CF strains were genotyped with flanking markers in order to map the regions of genetic heterogeneity, bringing this to a total of 105 microsatellite markers.

## Supplementary Material

Additional File 1 Click here for file
