# Supplementary material for: Spontaneous rescue from cystic fibrosis in a mouse model
Source: BMC Genet. 2006 Mar 29;7:18. doi: 10.1186/1471-2156-7-18 (PMC1448185; doi:10.1186/1471-2156-7-18)
Supplement: Additional File 1 [file 1471-2156-7-18-S1.PDF]

Additional File 1.

Marker genotypes of 105 microsatellites of F_{26} CF/1-*Cftr*$^{TgH(neoim)Hgu}$ and F_{26} CF/3-*Cftr*$^{TgH(neoim)Hgu}$ CF mice and the reference inbred strains C57BL/6, BALB/c, DBA/2J.

Marker [a]	Chr [b]	Locus [c] (MGI cM)	STS [d] (MGI Kbp)	*CF/1*	CF/3	DBA/2J	C57BL/6	BALB/c
D1Mit211	1	15	25851	b (146)	b (146)	b (146)	c (139)	a (149)
D1Mit303*§	1	34.8	64497	124/130	124/130	b (124)§	a (130)§	a (130)
D1Mit136	1	59.6	104108	a (108)	a (108)	b (104)	c (102)	a (108)
D1Mit450	1	79	157739	a (118)	a (118)	b (106)	a (118)	a (118)
D2Mit372	2	27.3	36375	b (119)	b (119)	b (119)	b (119)	a (127)
D2Mit37	2	45	75088	c (146)	c (146)	a (182)	b (174)	c (146)
D2Mit525	2	61.2	131827	a (127)	a (127)	c (117)	0	b (125)
D2Mit285	2	86	153600	0	b (150)	d (138)	a (152)	c (148)
D3Mit130§	3	3.9	10319	a (140)	a (140)	c (121)§	b (123)	c (121)§
D3Mit96§	3	23.3	48811	b (221)	b (221)	a (225)§	c (211)	a (225)§
D3Mit156	3	45.2	95440	c (254)	c (254)	a (266)	d (252)	b (260)
D3Mit254	3	64.1	135645	a (150)	a (150)	c (128)	a (150)	b (138)
D3Mit163	3	87.6	160819	a (147)	a (147)	a (147)	0	b (146)
D4Mit55	4	19.8	45000	a (184)	a (184)	c (164)	a (184)	b (180)
D4Mit116§	4	40	82509	c (136)	c (136)	a (152)	c (136)	b (144)
D4Mit169	4	58	122413	b (103)	b (103)	b (103)	a (105)	b (103)§
D4Mit310	4	71	146810	c (117)	c (117)	b (121)	c (117)	a (127)
D5Mit193	5	1	4246	b (136)	b (136)	a (147)	b (136)	c (134)
D5Mit107	5	26	45704	c (133)	c (133)	c (133)	b (136)	a (145)
D5Mit205§	5	45	94503	a (149)	a (149)	b (133)	a (149)§	a (149)§
D5Mit43§	5	83	-	a (138)	a (138)	b (132)§	b (132)§	b (132)§
D6Mit50	6	3.3	17149	c (164)	c (164)	c (164)	a (170)	b (168)
D6Mit351	6	20.4	47887	b (112)	b (112)	a (122)	b (112)	a (122)
D6Mit209	6	32	76629	c (130)	c (130)	a (138)	b (134)	c (130)
D6Mit69	6	35.15	84773	c (144)	c (144)	a (168)	b (164)	c (144)
D6Mit263	6	37	90434	b (144)	a (168)	a (168)	b (144)	b (144)
D6Mit284*	6	37.50	94143	136/142	136/142	c (136)	a (145)	b (133)

D6Mit67	6	41.50	99407	a (158)	a (158)	d (148)	c (152)	b (156)
D6Mit108	6	48.10	113086	a (141)	a (141)	c (129)	b (131)	c (129)
D7Mit152[§]	7	1	-	b (130)	b (130)	d (123)[§]	c (127)	a (133)[§]
D7Mit229	7	23	-	a (143)	a (143)	a (143)	b (139)	c (123)
D7Mit216	7	44	79835	b (184)	b (184)	a (186)	c (180)	b (184)
D7Mit330	7	57.5	111637	b (124)	b (124)	a (134)	b (124)	a (134)
D7Mit109[§]	7	66	128316	a (120)	a (120)	c (108)	b (118)[§]	b (118)[§]
D8Mit143	8	8	22746	a (279)	a (279)	b (269)	a (279)	a (279)
D8Mit289	8	11	27753	a (158)	a (158)	d (148)	c (152)	b (156)
D8Mit4	8	14	31006	d (157)	b (197)	c (195)	d(157)	a (200)
D8Mit24	8	18	33904	a (168)	a (168)	a (168)	b (160)	0
D8Mit204	8	20	-	a (150)	a (150)	a (150)	b (148)	a (150)
D8Mit65	8	22.5	42631	a (280)	a (280)	b (234)	b (234)	a (280)
D8Mit102	8	37	82331	c (117)	c (117)	b (118)	a (124)	c (117)
D8Mit318	8	57	115533	b (128)	b (128)	d (118)	c (124)	a (132)
D8Mit326	8	72	129107	b (125)	b (125)	a (127)	c (123)	b (125)
D9Mit250	9	5	8414	a (127)	a (127)	b (123)	b (123)	b (123)
D9Mit191[§]	9	26	48447	a (153)	a (153)	c (99)[§]	a (147)	c (99)[§]
D9Mit270	9	43	78643	c (140)	c (140)	b (144)	a (146)	c (140)
D9Mit137	9	66	118644	b (131)	b (131)	a (145)	a (145)	b (131)
D10Mit246[§]	10	5	12647	c (180)	c (180)	b (182)[§]	a (190)[§]	c (180)[§]
D10Mit250	10	19	27448	b (124)	b (124)	a (125)	a (125)	b (124)
D10Mit251[§]	10	21	28817	d (128)	e (120)	c (138)	a (144)[§]	b (140)
D10Mit107[§]	10	26	45181	b (105)	a (107)	b (105)[§]	b (105)[§]	a (107)
D10Mit36	10	29	50395	c (138)	c (138)	a (148)	b (146)	c (138)
D10Mit61	10	32	68764	a (148)	a (148)	a (148)	0	a (148)
D10Mit261	10	47	88037	a (114)	a (114)	d (104)	b (110)	c (108)
D10Mit103	10	70	128936	b (144)	b (144)	c (142)	d (140)	a (146)
D11Mit2	11	2.4	12217	b (122)	b (122)	a (124)	b (122)	c (112)
D11Mit271	11	21	45443	a (120)	a (120)	a (120)	a (120)	b (116)
D11Mit164	11	32	56598	b (121)	b (121)	b (121)	a (123)	b (121)
D11Mit208	11	33	58198	b (123)	b (123)	b (123)	a (129)	b (123)
D11Mit350	11	34.45	62866	b (96)	a (102)	b (96)	a (102)	b (96)
D11Mit4*	11	37	68181	d (244)	244/310	b (300)	c (246)	e (242)
D11Mit90	11	42	70071	c (150)	b (152)	a (176)	c (150)	b (152)
D11Mit322	11	44	76151	c (107)	b (109)	b (109)	a (113)	b (109)
D11Mit99*[§]	11	59.5	99232	c (120)	120/123	c (120)[§]	a (124)	b (123)[§]
D11Mit49	11	77	117695	b (156)	b (156)	b (156)	a (158)	a (158)
D12Mit37	12	1	5391	a (140)	a (140)	a (140)	a (140)	b (118)
D12Mit153	12	15	29938	a (160)	a (160)	b (158)	d (142)	c (156)

D12Mit159	12	38	79852	a	a	a	a	a
D13Mit3	13	10	-	0	0	a (196)	c (159)	b (188)
D13Mit224	13	37	62991	b (95)	b (95)	b (95)	a (117)	b (95)
D13Mit194	13	49	96051	c (146)	c (146)	a (152)	b (150)	c (146)
D13Mit263	13	71	-	a (128)	a (128)	a (128)	a (128)	b (122)
D14Mit11[§]	14	0.7	7425	a (158)	a (158)	a (158)	b (152)[§]	b (152)[§]
D14NDS1	14	2.5	15722	b (196)	c (190)	a (201)	d (182)	c (190)
D14Mit202	14	7.5	21569	a (146)	b (144)	a (146)	a (146)	b (144)
D14Mit44	14	10	22775	a (150)	b (148)	a (150)	a (150)	b (148)
D14Mit254	14	12	26218	a (122)	b (108)	a (122)	a (122)	a (122)
D14Mit234	14	22.5	50796	a (131)	a (131)	d (113)	b (127)	c (121)
D14Mit155	14	25	50981	c (201)	c (201)	a (210)	c (201)	b (205)
D14Mit262*	14	28.4	-	154/158	c (154)	c (154)	d (124)	b (156)
D14Mit237	14	32.5	61498	b (125)	a (134)	b (125)	b (125)	b (125)
D14Mit239	14	42.5	66468	c (125)	c (125)	a (131)	b (129)	c (125)
D14Mit106	14	48	92996	c (307)	b (309)	c (307)	a (312)	c (307)
D14Mit166*	14	52	-	128/144	a (144)	b (134)	a (144)	c (128)
D14Mit266*	14	60	114140	176/180	176/180	c (180)	a (148)	b (176)
**D15Mit102**	**15**	**6.7**	**66762**	**a (201)**	**a (201)**	**a (201)**	**a (201)**	**b (188)**
D15Mit100	15	21.1	51928	a (129)	a (129)	c (113)	d (111)	b (127)
D15Mit61	15	26	59669	a (100)	a (100)	b (98)	a (100)	a (100)
D15Mit46	15	27.5	62161	c (142)	d (140)	a (152)	b (150)	e (138)
D15Mit270	15	28.4	64939	a (200)	a (200)	b (188)	a (200)	c (180)
D15Mit144	15	32.2	69798	c (127)	c (127)	a (145)	c (127)	b (143)
D15Mit70[§]	15	47.7	82663	a (152)	a (152)	b (144)[§]	a (152)	b (144)[§]
D15Mit35	15	61.7	105842	a (146)	a (146)	c (136)	b (142)	a (146)
D16Mit146[§]	16	16.9	23365	b (120)	b (120)	b (120)	a (123)[§]	a (123)
D16Mit76[§]	16	43	68471	a (108)	a (108)	a (108)[§]	b (89)	a (108)[§]
D17Mit117	17	29.4	49586	a (127)	a (127)	a (127)	b (123)	a (127)
D17Mit142*[§]	17	47.4	79676	123/128	c (123)	c (123)[§]	a (147)[§]	c (123)
D18Mit18[§]	18	2	-	b (142)	b (142)	a (144)[§]	a (144)[§]	c (140)
D18Mit110	18	4	11980	d (127)	a (153)	a (153)	b (149)	c (133)
D18Mit21	18	6	15706	c (134)	b (136)	c (134)	c (134)	a (140)
D18Mit34	18	12	-	c (128)	c (128)	a (138)	b (134)	c (128)
D18Mit54	18	26	53854	c (144)	c (144)	c (144)	a (148)	b (146)
D18Mit8	18	47	74859	a (77)	a (77)	a (77)	b (75)	c (71)
D19Mit68	19	6	3449	a (136)	a (136)	b (132)	a (136)	c (122)
D19Mit40	19	25	25012	a (112)	a (112)	b (106)	a (112)	c (102)

[a]Markers are abbreviated following the rules and guidelines of the International Committee on Standardised Genetic Nomenclature for Mice whereby DNA segments are symbolised according to the laboratory identifying or mapping the segment as “DNA segment, chromosome N, Lab name” and a serial number, where N is the chromosomal assignment (1-19) and is symbolised as DNLabcode.

[b]Chr refers to chromosome number.

[c]Locus refers to the map position of markers according to MGI genetic map.

[d]STS refers to the physical map position of markers.

Letters represent alleles. Allele size is in alphabetical order with (a) used for the longest allele. Fragment size in basepairs is indicated in parenthesis. Triple black lines separate chromosomes. Six alleles represented by 0 did not amplify. D12Mit159 allele size could not be unequivocally assigned to any of the five observed alleles. Heterozygosity was documented for eight markers (*). For 19 markers ([§]) the observed allele size generated by the specific published primer sets for C57BL/6, DBA/2J and BALB/c was discordant with the published allele sizes. (Adapted from ref. [18]).
